# Does the size of an object containing dental implant affect the expression of artifacts in cone beam computed tomography imaging?

**DOI:** 10.1186/s13005-022-00326-1

**Published:** 2022-06-29

**Authors:** Mahkameh Moshfeghi, Yaser Safi, Ingrid Różyło-Kalinowska, Shiva Gandomi

**Affiliations:** 1 Department of Oral and Maxillofacial Radiology, School of Dentistry, Shahid Beheshti University of Medical Sciences, Tehran, Iran; 2 Department of Dental and Maxillofacial Radiodiagnostics, Medical University of Lublin, Lublin, Poland; 3 Department of Oral and Maxillofacial Radiology, School of Dentistry, Ahvaz Jundishapur University of Medical Sciences, Ahvaz, Iran

**Keywords:** Artifacts, Cone beam computed tomography, Dental implant, Image quality

## Abstract

**Background:**

Artifacts fault image quality but handling several factors can affect it. This study was conducted to investigate the effect of object size on artifacts in cone-beam computed tomography systems.

**Methods:**

Five phantoms, each containing a titanium implant in a sheep bone block, were fabricated of various sizes ranging from XS to XL: The M phantom was the same size as the device’s field of view (FOV). The L and XL phantoms were 20 and 40% larger than the FOV while the S and XS phantoms were 20 and 40% smaller than FOV, respectively. Ballistic gelatin was used to fill the phantoms. Phantoms were scanned by NewTom VGI and HDXWill Q-FACE. The mean and standard deviation (SD) of gray values in each 120 ROI was obtained by OnDemand software. The contrast to noise ratio (CNR) was also calculated.

**Results:**

The gray value in S and M phantoms were more homogenous. The lowest SD value (10.20) was found in S phantom. The highest value for SD (125.16) was observed in XL phantom. The lowest (4.47) and highest (9.92) CNR were obtained in XL and S phantoms, respectively. HDXWill Q-FACE recorded a higher SD and a lower CNR than NewTom VGI (*P* < 0.05).

**Conclusion:**

Object dimensions of the FOV size or up to 20% smaller provided better image quality. Since the dimensions of soft tissue in most patients are larger than the selective FOV, it is recommended that in CBCT artifacts studies, an object with dimensions closer to the patient’s dimensions be used to better relate the results with the clinical condition, because the sample dimensions affect the amount of artifacts.

## Background

Radiography is important in management of dental diseases [1]. Dental implants are extensively applied for replacing missing teeth, because they help maintain bone structure and restore the occlusal function [2]. Patients who have undergone successful implant therapy must receive individualized, systematic and continuous supportive care for the peri-implant tissues [3]. Cone beam computed tomography (CBCT) is a three-dimensional imaging used in dentistry and is considered as one of the best imaging options for diagnosis [4, 5]. It not only provides appropriate visualization of small bony structure details, but also allows for the precise assessment of bone defects in all three dimensions [6]. The CBCT has a higher spatial resolution, smaller radiation dose, lower cost, and is more compact than MDCT [7]. CBCT has been successfully used in branches of dentistry such as endodontics, orthodontics and oral surgery [7]. Despite these advantages, CBCT has limitations such as metal streak artifacts that influence the quality of CBCT images. Artifacts, known as any error in the image that is not associated to the subject, are the main cause of image quality reduction [8].

Metal artifacts are the most common factors causing faults in the CBCT image diagnostic quality by decreasing the contrast of the image and obscuring essential sections and adjacent structures for the implant site [9]. Metal artifacts are produced by high density objects primarily as a result of beam hardening, as well as other factors like scatter and noise [10]. Beam hardening is a phenomenon that occurs when the x-ray beam travels from an object and the low-energy photons are absorbed more than the high-energy photons. Depending on the amount of X-ray absorption and further radiation attenuation due to the presence of a high density material, artifacts are most commonly presented as radiopaque and radiolucent strips, or streak lines [11]. The artifact intensity relies on the atomic number, position, and number of metal objects in the field of view (FOV) [12]. Several variables are involved in the occurrences of artifacts, such as exposure conditions, pixel size, and FOV [13]. Other factors such as beam quality and quantity, filter type, and rotation arc also influence gray value, noise, contrast, resolution, and artifacts [14, 15].

Considering the importance of dental implants for edentulous regions and the effects of artifacts on the image quality, the purpose of this study is to see how the size of an object of interest, containing dental implant, affects the CBCT artifacts.

## Methods

This in vitro descriptive-analytical cross-sectional study was conducted with twenty scans of five imaging samples using two CBCT units.

### The preparation of samples

This study was conducted on CBCT images of an implant placed in bone cube introduced subsequently into five different sized custom-made phantoms. Cylindrical phantoms with various sizes containing a bone block, filled with ballistic gelatin which simulated soft tissues. The body of the phantoms was made of thin plastic. The dimensions of the phantoms were measured by a caliper (Mitutoyo, Japan). All the phantoms were 8 cm high with various widths, so the horizontal dimensions of the phantoms were adopted to define their sizes. The first phantom labelled as medium sized (M) was fabricated with diameter of selected FOV in CBCT (8 × 12 cm) and used as a base for fabrication of other phantoms. The second and third phantoms were 20 and 40% larger than FOV and labelled as large (L) and extra-large (XL) phantoms, respectively. The fourth and fifth phantoms were 20 and 40% smaller than FOV, and labelled as small (S) and extra-small (XS), respectively. The dimensions of L phantom were similar to bizygomatic sizes in adults (8 × 14.5) [16]. The XL, S, and XS phantoms were of the following dimensions, respectively: 8 × 17, 8 × 9.5 and 8 × 7.

Using a milling machine with water cooling, a cuboid block of sheep mandible bone was prepared with dimensions of 10 mm width, 10 mm length, and 12 mm height. The center region of the top of the bone block was marked. Titanium implants (BioHorizons® Tapered HD dental implant) of 7.5 mm long with 4.5 mm diameters were placed into the prepared cavities inside the bone block using the BioHorizons Commercial kit and with the help of a periodontist. The central region on the bottom of the phantom was measured using a caliper and then marked. The bone block containing the implant was subsequently placed in center region of the five phantoms and fixed with silicon molding material. After bone block placement, the phantom was filled by ballistic gelatin made as described in previous studies [17]. The bone block was removed after each scan and positioned into the central regions of other phantoms. To standardize the region of interest (ROI) in images, three aluminum makers were used with length of 3 mm on three surfaces of the bone block for equalization of axial sections in sagittal and coronal planes.

### CBCT examination setup

The scans were obtained by an experienced radiology technician using the New Tom VGI CBCT scanner (Quantitative radiology, Verona, Italy) and HDXWill Q-FACE CBCT scanner (Seoul, South Korea) with exposure parameters for a standard patient as recommended by the manufacturer. The exposure settings of New Tom VGI scanner were 110 kVp and 3.3–10 mA. The HDXWill Q-FACE scanner was used with the fixed exposure settings of 100 kVp and 4 mA. Both devices were chosen with the FOV dimensions of 8 × 12, so the size of FOV was fixed in different scans. Metal artifact reduction (MAR) was not activated in any of the devices. The phantoms were positioned in the central region of the FOV using the CBCT scanner’s laser indicator lights in three planes (X, Y and Z axes) and further confirmed on scout views. Each scan was taken twice by each CBCT unit for all the phantoms. The difference between the received images was insignificant (< 5.00%) for mean gray value (MGV) and standard deviation of gray values (SD). In the case of discrepancy, the mean for the obtained images was defined as sample.

**Fig. 1:**
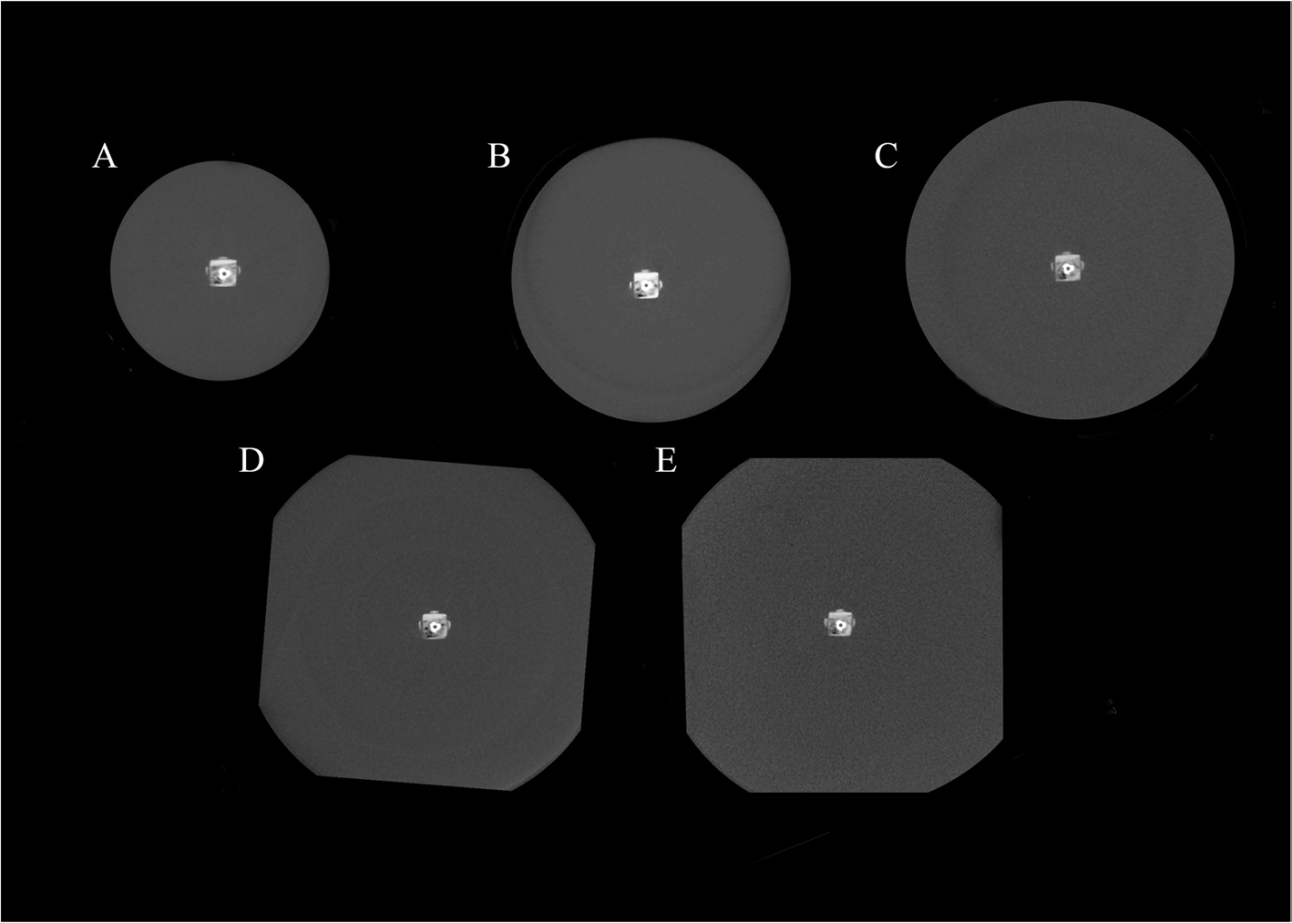
The axial images prepared by NewTom VGI. **A** XS phantom, **B** S phantom, **C** M phantom, **D** L phantom, and **E** XL phantom

**Fig. 2:**
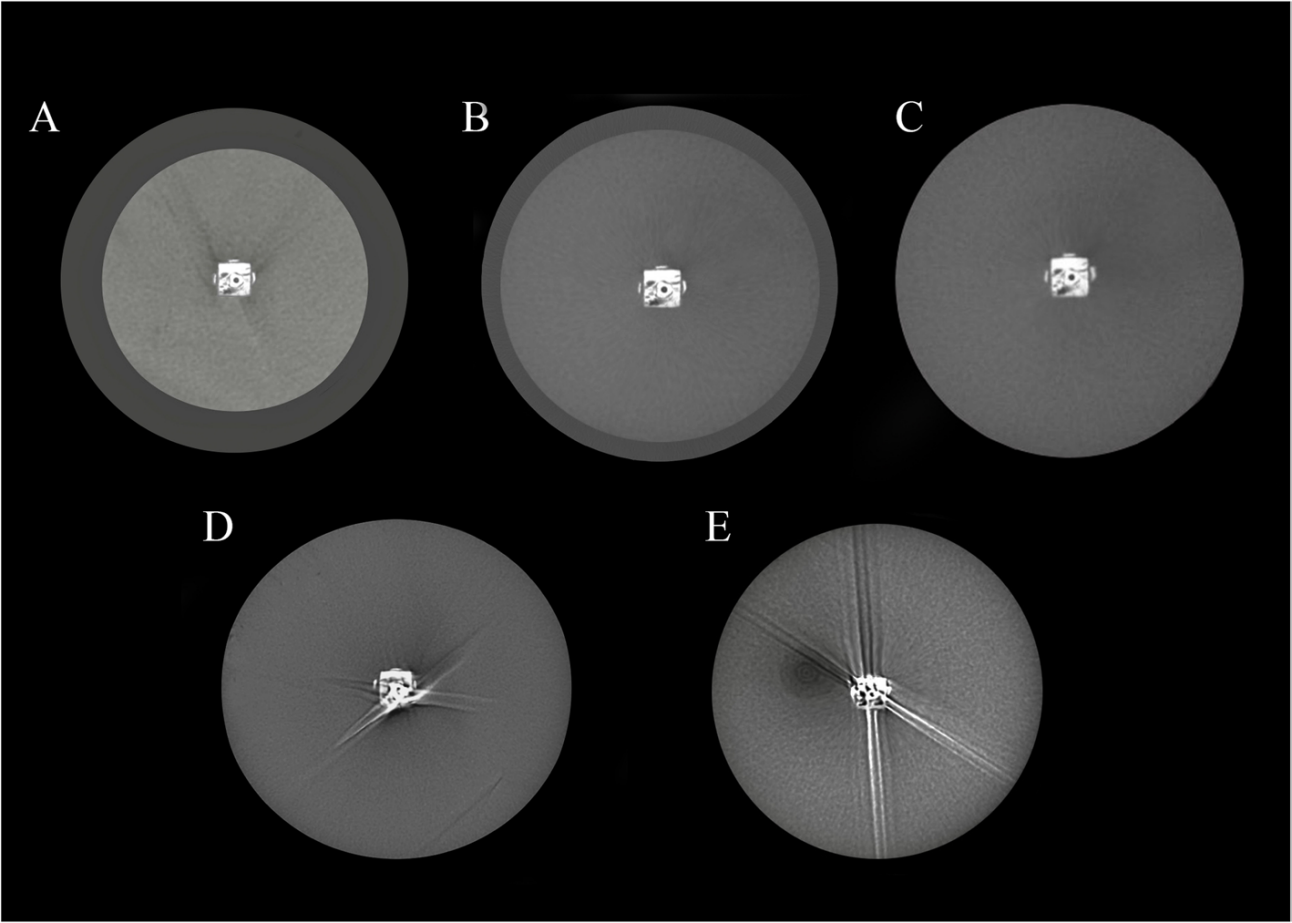
The axial images prepared by HDXWill Q-FACE. **A** XS phantom, **B** S phantom, **C** M phantom, **D** L phantom, and **E** XL phantom

The scans prepared by New Tom VGI CBCT scanner and HDXWill Q-FACE are presented in Figs. 1 and 2.

### CBCT volume assessment

**Fig. 3:**
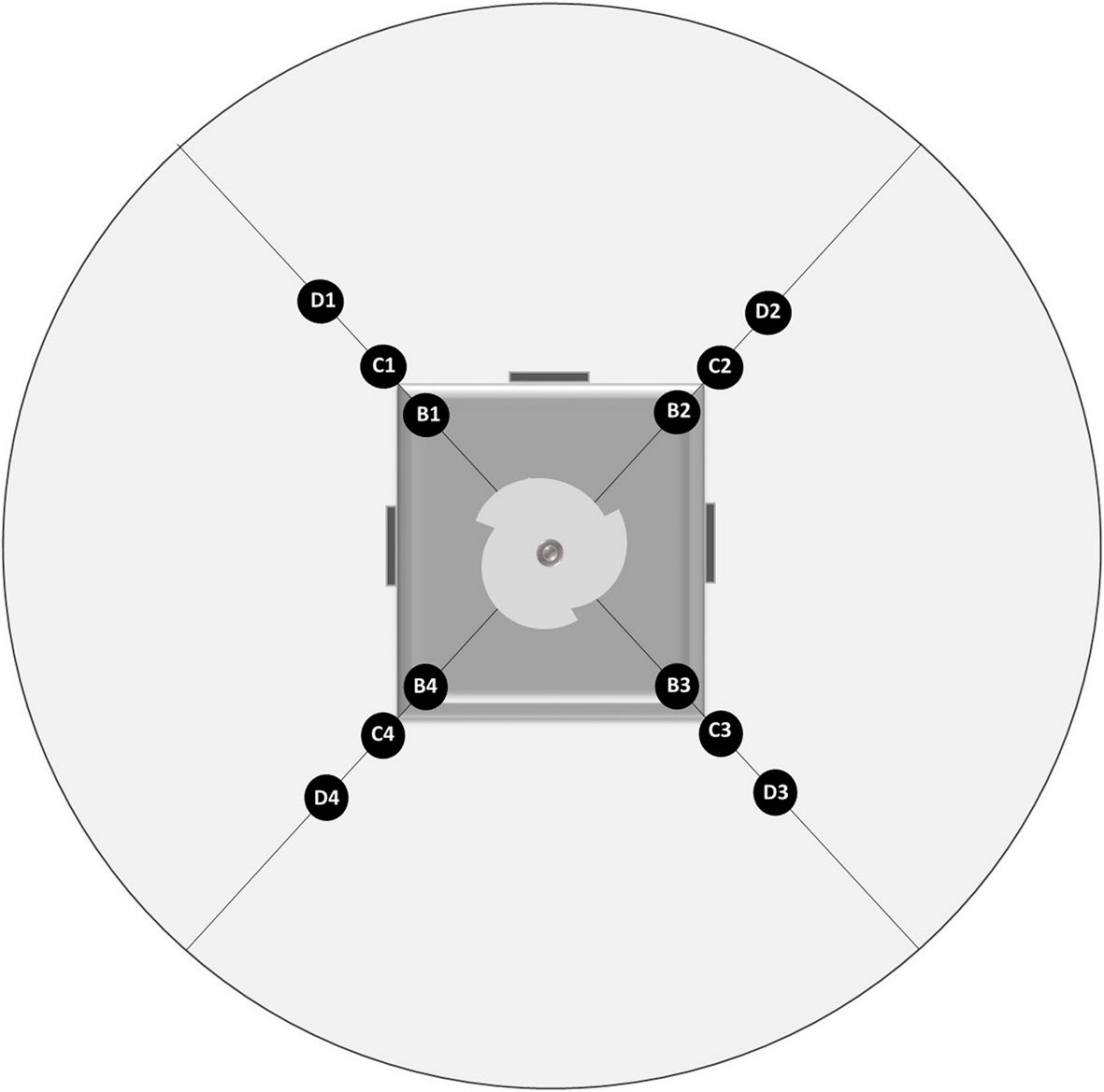
A schematic view of ROIs in axial section. White circle represents gelatin filled phantom. Gray rectangle shows bone cube with an implant placed in its center. Dark gray rectangles at three surfaces of bone cube represent aluminum markers. Black circles show 12 ROIs

OnDemand 3DApplication software (Cybermed Inc., Seoul, Korea, version 1) was used to reconstruct scans with voxel size of 0.1 mm in a standard arrangement of Digital Imaging and Communications in Medicine (DICOM). The OnDemand software was used to measure artifacts. All the investigations were conducted by 16-bite images. Circles with 2 mm diameters were used as ROI by using histogram tools in OnDemand software. Twelve ROIs were selected in four directions on bone block diameters. These diameters traverse from central region of implant (Fig. 3). ROIs were (1) four points inside bone and 2 mm from external bone surface (B), (2) four points inside gelatin, tangent on external surface of bone, without including bone tissue (C), (3) four points inside gelatin, 4 mm from external surface of bone (D). In other words, B ROIs were located within the bone tissue, C ROIs were the gelatin areas closest to the implant as the metal artifact producing object, and D ROIs were further away.

The MGV and SD were recorded in each ROI. The SD shows image noise and presence of artifacts. To standardize measurement for CBCT units, contrast to noise ratio (CNR) was used, as follows [18]. To calculate contrast, the average MGV of the gelatin ROIs as a low radiodensity substance was subtracted from the MGV of the bone ROIs, as a high radiodensity tissue for each phantom. Mean SD of ROIs in gelatin was considered as noise.

$$\varDelta \delta ={MGV}_B- MGV\frac{}{c,d}\kern3em a= SD\frac{}{c,d}$$

All CBCT scans were evaluated by a calibrated oral and maxillofacial radiologist. In this study, the subjectivity of the ROI selecting was the source of the bias. The intra-observer agreement was calculated to confirm the reliability. For this purpose, all the MGV and SD records were evaluated twice at 2-month intervals using ICC (intraclass correlation) test.

### Data analysis

The data was analyzed by SPSS software (version of 26) and descriptive data was quantified as mean ± SD. The data was investigated for normality using the Shapiro-Wilk test. Since the data was normal, two-way and three-way ANOVA pathway were used and *P* < 0.05 was considered as significant. Multiple comparison Bonferroni correction was used for comparing phantom sizes and ROI locations. To compare phantom sizes, SD differences were measured only in the gelatin region.

The MGV for 120 points were analyzed by Adobe photoshop software (22.3.1 version). To define color, highest value was assigned for white color, and lowest value for black color.

## Results

### Intra-operator reliability

Measurements for the first and second replicates were recorded, and intra-class correlation coefficients (ICC) were established for all measurements. Most measures demonstrated a high degree of reliability between the first and second replicates with ICC values exceeding from 0.83 to 0.95.

### Mean gray value

**Fig. 4:**
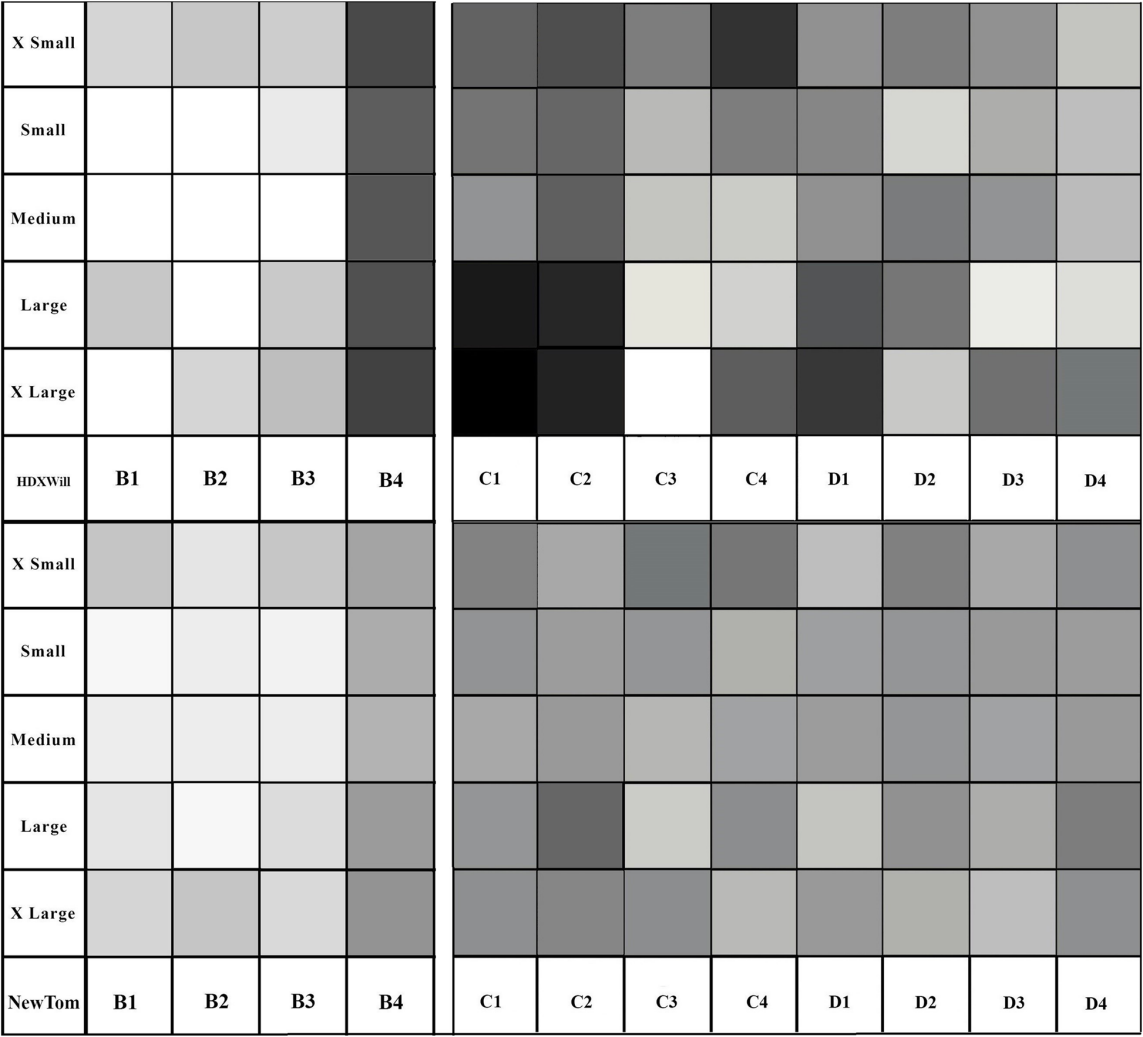
The results for MGV of ROIs in bone (left), and gelatin (right). Adobe photoshop software was used to make the image, which depicts the numerical gray value of each 120 measured ROIs as various shades. Therefore, each cell represents a gray tone similar to the CBCT image. ROIs are represented by columns, and phantom sizes are represented by rows. The cells at the top of the image are related to the HDXWill Q-FACE device, whereas cells at the bottom shows the results of the NewTom VGI device. To make it easier to distinguish between different shades, the ROIs values in gelatin (C and D) and bone (B) were defined separately

The value range for ROI in gelatin (locations C and D) and bone location (B) was defined separately (Fig. 4). Gray shadows have a significant similarity in NewTom compared to HDXWill. Gray shadows in gelatin region of small and medium phantom were more homogenous.

### The overall SD

The lowest SD value (8.90) was found in S phantom, D location and NewTom VGI CBCT. The highest value for SD (436.87) was observed in XL phantom, B location and HDXWill Q-FACE CBCT.

The three-way ANOVA analysis of the main effects and interactions between CBCT units, phantom sizes, and ROI location on SD showed significant effects for ROI location (*P* = 0.000), phantom size (*P* = 0.000), CBCT units (*P* = 0.000), ROI location×phantom size (*P* = 0.000), phantom sizes×CBCT (*P* = 0.000), and ROI location×Phantom sizes×CBCT (*P* = 0.000). The most notable effects were related to phantom size (Eta = 0.765), and ROI location (Eta = 0.760).

**Fig. 5:**
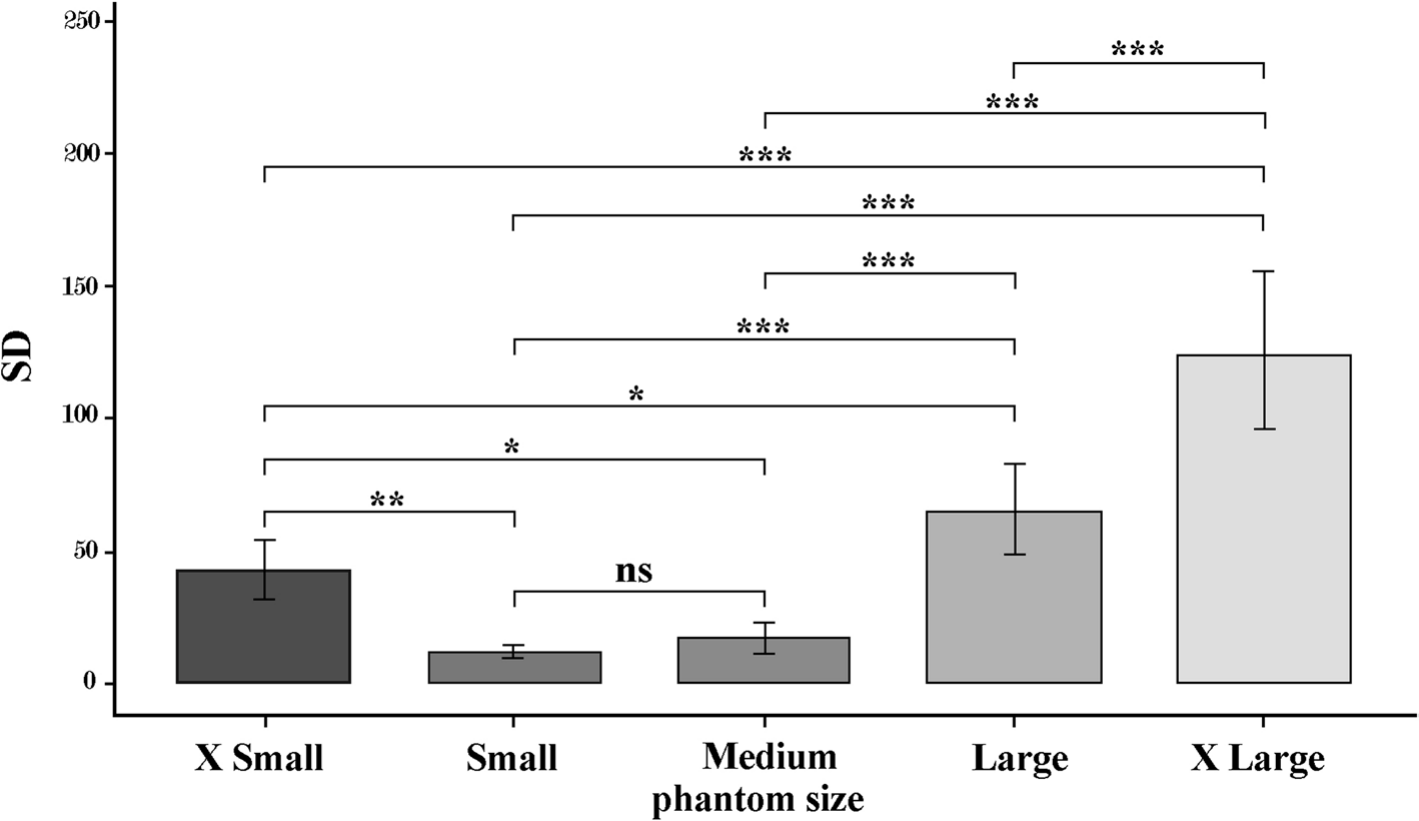
The SD in different phantom sizes (ns: not significant, *: *p* < 0.05, **: *p* < 0.01, ***: *p* < 0.001)

The results for Multiple comparison Bonferroni correction for comparison of phantom sizes showed significant differences between all the phantoms (*P* < 0.05) except between small and medium phantoms (*P* = 1.00). It should be mentioned that to compare SD between different phantom sizes, this parameter was measured in ROI C and D. The lowest (10.20) and highest (125.16) SD were obtained in S and XL phantom, respectively. The biggest difference was observed between small and X-large (113.20). The values for SD in phantom sizes are shown in Fig. 5.

**Fig. 6:**
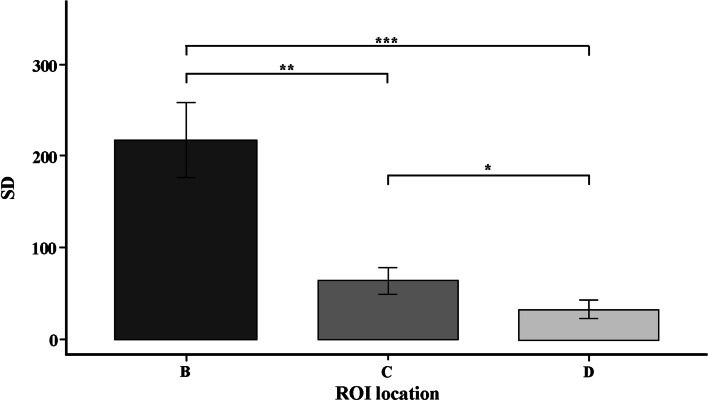
The SD in different ROI location (*: *p* < 0.05, **: *p* < 0.01, ***: *p* < 0.001)

**Fig. 7:**
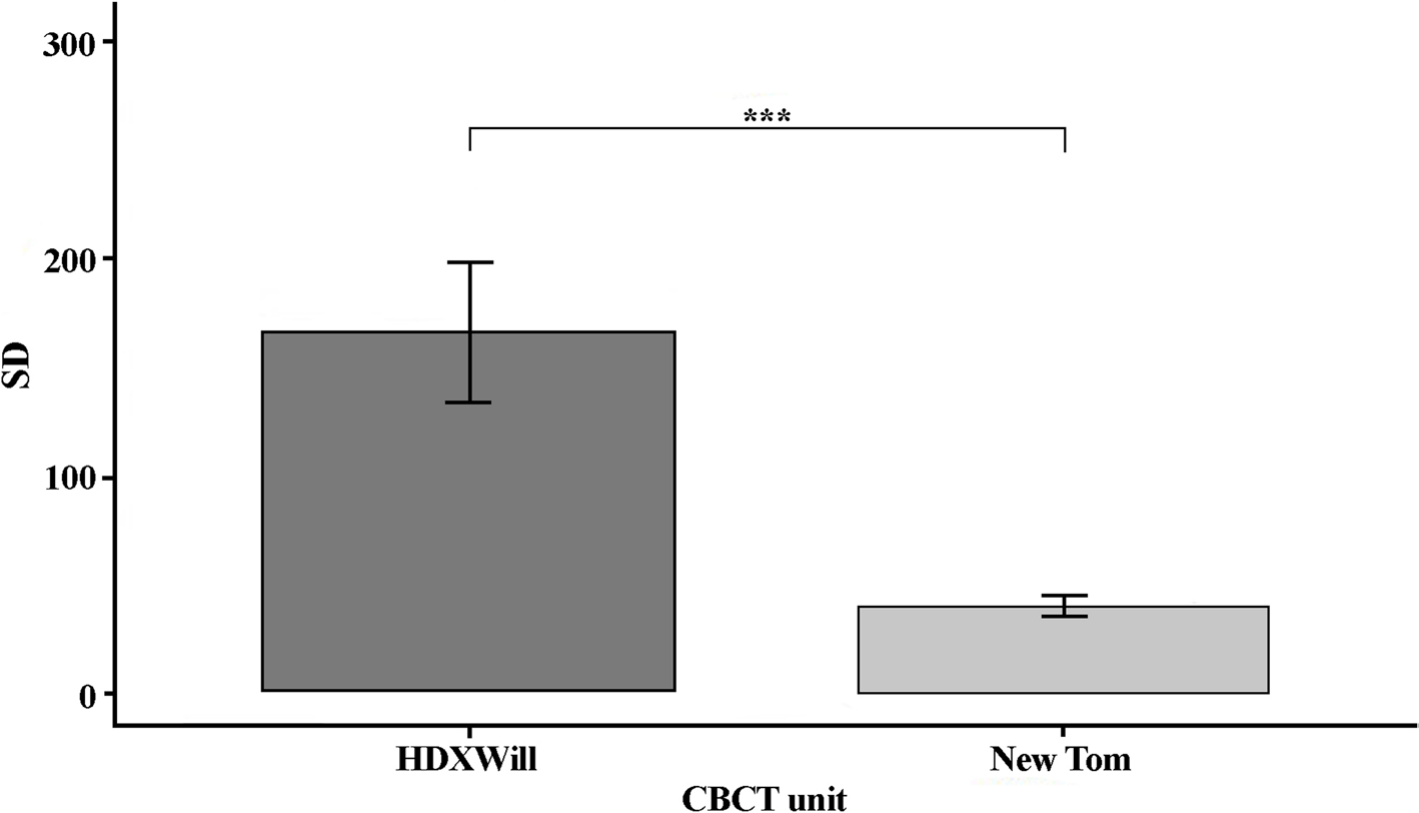
The SD in different CBCT units (*: *p* < 0.05, **: *p* < 0.01, ***: *p* < 0.001)

The results for artifacts levels in ROI locations and CBCT units are shown in Figs. 6 and 7. ROI location showed significant differences between B, C and D locations. The results showed fewer artifacts in NewTom compared to HDXWill CBCT (*P* = 0.03).

### The overall SNDR

The two-way ANOVA analysis results of the main effects of CBCT units and phantom sizes and their interactions based on SNDR showed significant effects for phantom size (*P* = 0.000) and CBCT units (*P* = 0.003). No statistically significant differences were observed for phantom sizes×CBCT unit (*P* = 0.795). The most notable effects were related to phantom size (Eta = 0.896).

**Fig. 8:**
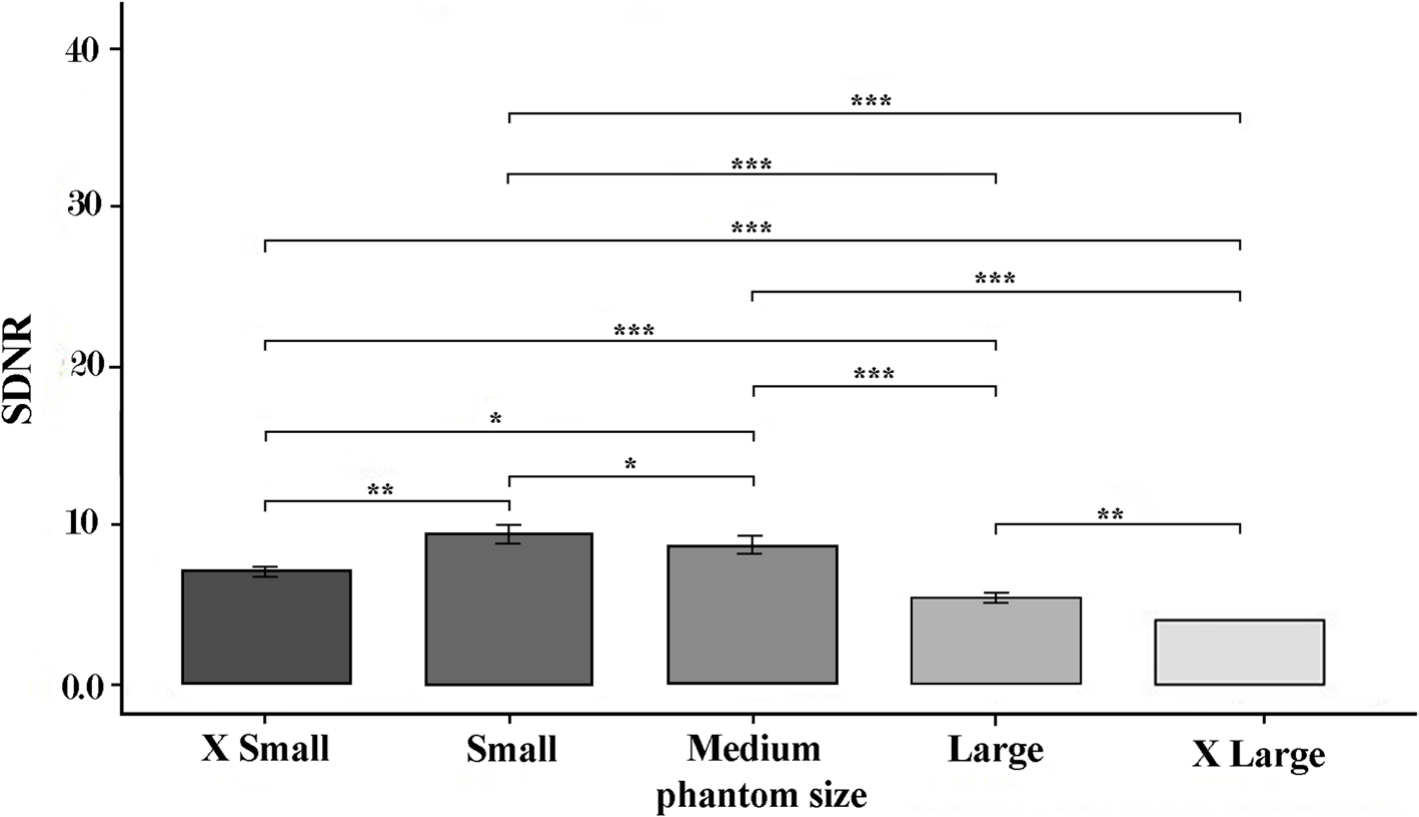
The CNR in different phantom sizes (*: *p* < 0.05, **: *p* < 0.01, ***: *p* < 0.001)

The results for comparing Bonferroni correction between CNR and phantom size showed significant differences between all the phantoms (*P* < 0.05). The lowest (4.47) and highest (9.92) CNR values were obtained in XL and S phantom, respectively. The most notable difference was observed between small and X-large (5.45). Figure 8 illustrates the mean for phantom sizes.

**Fig. 9:**
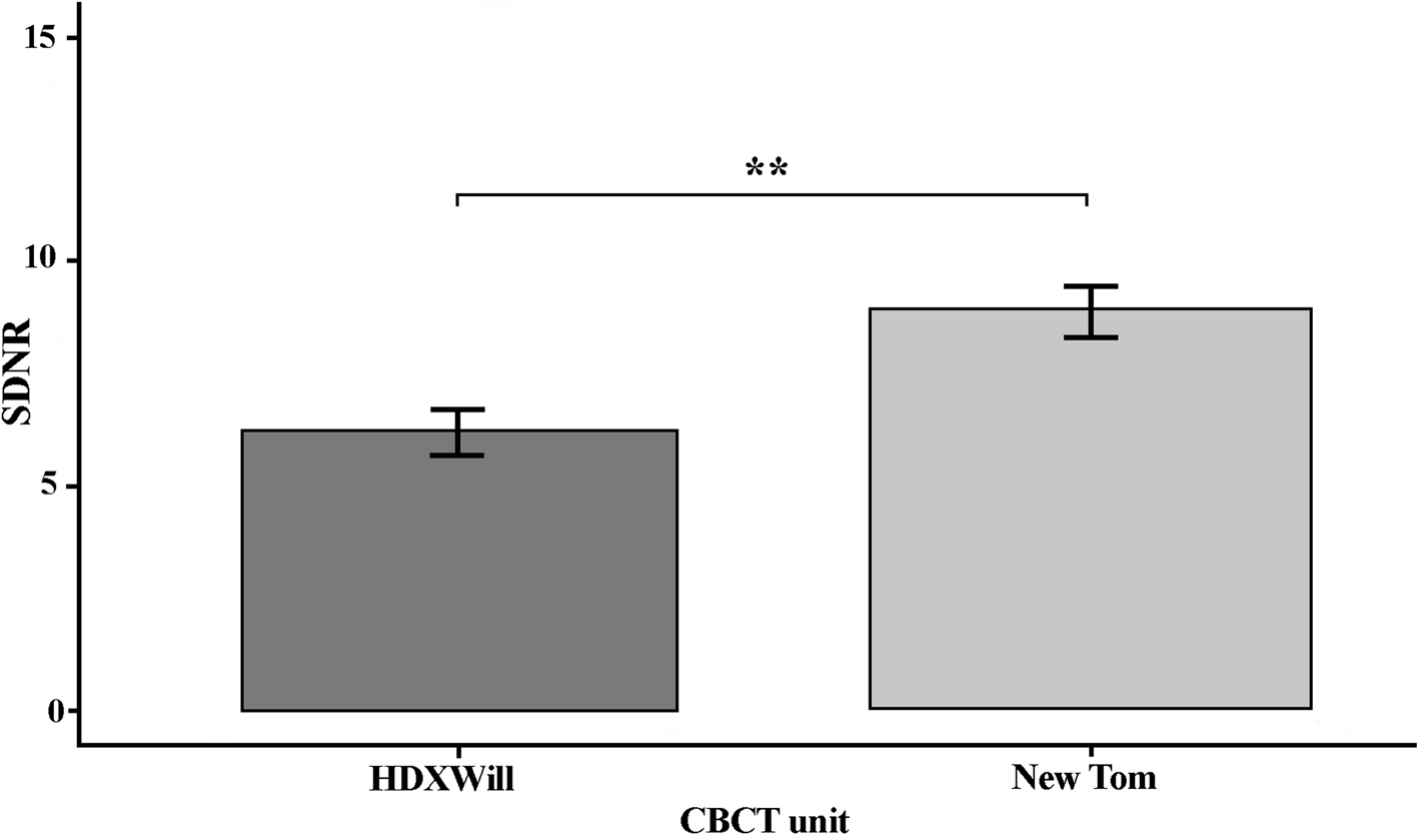
The CNR in different CBCT units

The results for CNR levels in CBCT units are shown in Fig. 9. The results show significant difference between HDXWill and NewTom (*P* = 0.008).

## Discussion

Metal artifacts produced by dental implants influence the image quality of anatomic structures and complicate bone assessment for adjacent implants. Many studies have investigated artifacts in CBCT, but there are issues in quantifying artifacts based on image quality [19]. The qualitative assessment, based on the observer judgement, is helpful in evaluating the decreased effects of artifacts and diagnosis, but this assessment cannot compare the efficiency of systems and cannot be used in quality control.

It’s difficult to provide an objective and quantitative assessment of artifacts in CBCT [20]. There are no standard parameters to determine the quantity of artifact effects on voxel [21]. Studies have evaluated artifacts by various parameters, such as MGV and SD of gray values, and CNR [22]. The MGV approximates the radiopacity and radiolucency produced by metals. Higher SD represents more noise and lower image quality, while CNR shows effect of artifacts on image contrast and helps compare CBCT units. A higher value for CNR shows better image contrast and quality [23]. Although SD and CNR provide valuable information, they require precise interpretation, because voxel sizes and intrinsic noises also affect image quality [24]. Furthermore, the gray value of a CBCT image depends on various factors such as exposure settings, the patient’s position, the device model, and the ROI’s area or size. This may have an impact on the results and impair their precision. According to recent studies, to reduce the undesirable effects of inherent noise, the amount of streak artifacts decreased by MAR was automatically counted in MATLAB using canny edge detection, which is an approach that is less dependent on the image’s gray value [20, 25].

Our results show that object sizes representing dimensions of patient soft tissue had significant effect on artifacts, such that objects equal to FOV and 20% smaller than FOV had more homogenous MGV, higher CNR and lower SD, resulting in less intense artifacts and better image quality. S phantoms produced better quality images than M phantoms, but differences were insignificant. Objects larger than the FOV had more intense artifacts. It has been proved that exomass, or objects outside the FOV that remain between the focal spot and the receptor, influenced image quality. Artifacts related with exomass directly influence voxel value and image noise [26]. To decrease artifacts, it is better to apply larger FOV in patients with larger size. It is essential to mention that increasing FOV size may decrease image resolution. Our findings are in agreement with results reported by Seet et al. who showed that increased phantom size influences CT value, while decreasing precision and image quality [14]. Previous studies have reported that beam scatter has a direct relation to object size, such that increased object size augments beam diffraction [27]. This result was confirmed in objects larger than FOV, however, in XS phantom, higher SD and lower CNR were detected compared to S and M sized objects. So, it can be assumed that decreased soft tissue volume doesn’t necessarily increase image quality. The existence of more artifacts in XS objects compared to S and M objects could be related to the fact that beam streaks does not cross over the borders of objects. Therefore, an artifact concentration could be expected in smaller sized objects [28]. Spread of beam streaks occurs in larger objects, further improving image quality around the metallic material. This issue could be applied to larger objects as long as it does not cause an exomass artifact by overextending the selected FOV. To prevent truncating the axial section data, manufacturers recommend using a FOV equal to the patient’s maximum dimensions. However, imaging with FOV equal to object dimensions also produces exomass artifacts. Very small parts of medium phantom were positioned in exomass on closer inspection. This shows CBCT sensitivity to exomass artifacts. A difference of 20 mm [2] in axial section is enough for producing artifacts resulting from exomass [14].

The results showed that ROI location also influences image quality. The highest SD was observed in areas of the trabecular bone that were intrinsically inhomogeneous. Knowing that artifacts cause appearance of regions with lower gray values near the implant has helped in preventing the diagnosis of false positive diagnoses of peri implantitis [29]. It was reported that trabecular microstructure parameters observed in micro-CT used for quantification of artifacts are more appropriate than SD of the gray values to evaluate artifacts in bones [22]. In our study, ROIs near the implant produced more SD. In other words, artifacts were higher in locations close to metal objects. Similar to our findings, previous studies have shown that distance has a significant effect on the production of artifacts, and increased distance decreases artifacts [11, 30, 31]. Mancini et al. investigated three ROI location (1.5, 2.5, and 3.5 cm) and demonstrated non-significant difference for the SD in distances of 1.5 and 2.5 cm, while the SD was lower in 3.5 cm location [32]. Fontenele et al. did not observe significant differences for artifacts and image quality in locations of 1.5, 2.5, and 3.5 cm [33].

The CBCT units had significant effects on the image quality. Although the main purpose of this study was to assess the effect of object size on the amounts of produced artifacts, we could also make a comparison between the two CBCT systems. However, the multi-faceted nature of these systems, such as hardening, Voxel size, voltage, the FOV and current intensity can affect the obtained results [34]. NewTom VGI unit were characterized by less SD and more CNR, therefore less artifacts were observed in NewTom VGI unit than HDXWill CBCT unit, which may be related to the higher kVp value of the Newtom VGI CBCT scanner. Furthermore, NewTom scanner has an automatic exposure system via “safebeam sensor” which will adapt itself to the patient’s factors. Probably, this is why by modifying mA in a NewTom device the image quality difference between phantoms of different sizes is much less than in a HDXWill device. The fact that mA, as an interfering variant, could not be fixed was a limitation of the present study. Codari et al. also showed that the NewTom VGI system has better diagnostic power than the Picasso Trio system due to its 360 degree rotation and increased data receiving ability [35]. Moreover, Kamburoğlu et al. showed lower beam hardening artifacts production in the NewTom VGI system than in the Promax system and suggested the use of NewTom VGI systems for patients with multiple restorations, prosthesis, and implants [29]. The differences in diagnostic value between different CBCT units are mainly attributed to their resolutions [36].

In our study, dental implants were placed in sheep mandible bone blocks to simulate in vivo conditions. We used ballistic gelatin to simulate soft tissue. Other studies have used water, ice, wax, ultrasound gel, and acrylic resin to simulate soft tissue [17, 37, 38]. A recently study has introduced ballistic gelatin as the best simulator of soft tissue [39]. Despite the inhomogeneous nature of human tissue, we used homogenous materials for evaluating the artifacts. Homogeneous materials have advantages such as allowing for more precision in the estimation of gray values. Most studies conducted on CBCT imaging are done in vitro due to ethical considerations. Because of FOV size and various exposure parameters, studies done on patient’s image data fail to provide accurate information for specific variables. In vitro studies, like this study, allow for precision in controlling interfering variables. Future studies can investigate voxel size, exposure parameters, high-density materials in other positions in dental arch, and an increased number of implants.

This study is an in vitro study and the data must be cautiously used under clinical condition because X-ray interferences are different in each patient [30]. In this study, the dental implant was positioned in central region of the FOV which may produce lower levels of noise compared to marginally positioned metal object [40]. This study has not examined the motion artifacts that can cause problems in clinical conditions. In addition, the exact effects of artifacts on trabecular structures were not investigated due to the intrinsic limitations of CBCT systems.

## Conclusion

In conclusion, object size has significant effects on image quality and artifacts. Objects equal to FOV and 20% smaller than FOV produced images with better quality and objects larger than the FOV had more intense artifacts. Firstly, since the dimensions of soft tissue in most patients are larger than the selective FOV, it is recommended that in CBCT artifacts studies, an object with dimensions closer to the patient’s dimensions be used to better relate the results with the clinical condition, because the sample dimensions affect the amount of artifacts and obtained results. Secondly, because the smaller object (XS) was of poorer quality, the presence of more soft tissue is expected to diminish the artifact, as long as it is not larger than FOV. Thus, developing a soft tissue simulator tool while using a larger FOV for specific diagnostic demands, may be recommended to reduce artifact. However, this recommendation requires further studies in this field to determine its clinical value and importance. Imaging with NewTom VGI CBCT scanners can be advised if a significant production of metal artifacts is expected in the image.

## Data Availability

The datasets used and/or analyzed during the current study are available from the corresponding author on reasonable request.
